# Shopping list development and use of advertisements’ pre-store food-buying practices within different socio-economic status areas in South Africa

**DOI:** 10.1108/BFJ-11-2016-0556

**Published:** 2017-12-04

**Authors:** Rodney Graeme Duffett, Crystal Foster

**Affiliations:** 1 Faculty of Business and Management Sciences, Cape Peninsula University of Technology, Cape Town, South Africa; 2 Department of Agricultural and Food Sciences, Cape Peninsula University of Technology, Cape Town, South Africa

**Keywords:** South Africa, Developing country, Pre-store food-buying practices, Shopping list development, Socio-economic status (SES) areas, Use of advertisements

## Abstract

**Purpose:**

The purpose of this paper is to determine whether there is a difference in the development of shopping lists and use of advertisements as pre-store food-buying practices in terms of planned shopping by South African consumers who dwell in different socio-economic status (SES) areas. The paper also considers the influence of shopper and socio-demographic characteristics on pre-store food-buying practices in a developing country.

**Design/methodology/approach:**

A self-administered questionnaire was used to survey 1 200 consumers in retail stores in low, middle and high SES areas in South Africa. A generalised linear model was employed for the statistical analysis of pre-store food-buying practices within the SES area groups in a developing country.

**Findings:**

South African consumers that reside in high SES area displayed the largest of shopping list development, while consumers who dwell in low SES areas showed the highest incidence of advertisement usage. Several shopper and socio-demographic characteristics were also found to have an influence on pre-store food-buying practices in different SES areas in South Africa.

**Research limitations/implications:**

A qualitative approach would offer a deeper understanding of consumers’ pre-store food shopping predispositions as opposed to the quantitative approach, which was adopted for this study. A longitudinal design would also provide a more extensive representation of pre-store food shopping practices over a longer time frame than cross-sectional research. The survey was conducted on Saturdays, whereas consumers who shop during the week may have different shopping and socio-demographic characteristics.

**Practical implications:**

Astute food brands, marketers and grocery stores could use the findings of this study to assist with their marketing efforts that they direct at consumers in different SES areas in South Africa and other developing countries.

**Social implications:**

The findings of this study may assist consumers in developing countries, especially those who reside in low SES areas, with food-buying strategies to reduce food costs, make wiser purchase decisions and reduce shopping.

**Originality/value:**

No study (to the best of the researchers’ knowledge) has considered shopping list development and use of advertisements’ pre-store food-buying practices in different SES areas in a developing country. Furthermore, there is a dearth of research analysing shopper and socio-demographic characteristics in relation to pre-store food-buying practices among different SES areas in developing and developed countries.

## Background

South African consumers have experienced increasing debt levels over the past decade as a result of an enduring recession. One of the many consequences of this economic downturn has been a major increase in energy and transport, which, coupled with the current drought experienced Africa, means that food prices have markedly increased in South Africa (South African Reserve Bank, 2016). The Community Survey 2016 reported that one in five households did not have sufficient money to buy food in the past 12 months (Statistics South Africa, 2016a). Hence, consumers generally agree that their economic situation has an important effect on their shopping behaviour (Darko *et al.*, 2013; Petzer and De Meyer 2013). Food-buying practices can be used by consumers to help to reduce the amount of money, which is spent on food, and increase funds that are available for other household expenses.

Despite the high rate of unemployment and household indebtedness, consumer confidence and subsequently household expenditure on consumer goods and services has been on the increase (South African Reserve Bank, 2016). However, consumers should consistently employ effective purchasing strategies to protect themselves against uncertain economic times, especially since consumer credit facilities are less freely available owing to the National Credit Act (Hampson and McGoldrick, 2013; O’Haughey, 2016). The National Agricultural Marketing Council (2016) reports that South Africa has one of the highest inflation rates for food in comparison to other countries, with the cost of an urban food basket increasing by 16.3 per cent over a period of one year (July 2015-July 2016).

It is generally accepted that there is an inverse relationship between income level and the amount of money, which is allocated to food purchasing. Hence, low-income households usually devote a larger percentage of their total expenditure to food, while in each successively high-income group the amount that is spent on food declines as a percentage of total expenditure (Frazao *et al.*, 2007; Darmon and Drewnowski, 2015; KPMG, 2016). South African consumers, particularly from low-income households, are impacted the most by rising food costs and currently spend 57.1 per cent (compared to 49.1 per cent of July the previous year) of their disposable income on food compared to the 2.3 per cent of high-income consumers (National Agricultural Marketing Council, 2016). Therefore, consumers from low socio-economic groups often have to cut back on food spending to make room for other essentials such as housing and utilities (Ward *et al.*, 2012). Socio-economic status (SES) differences in household food purchasing behaviour were investigated by Turrell *et al.* (2004), who found that residents of socio-economically disadvantaged areas have purchasing patterns that are different compared to those in more advantaged areas. Ellaway and Macintyre (2000) agree that shopping practices differ by neighbourhood and income group.

The use of consumers’ money-saving techniques, particularly during difficult economic times, has not been extensively researched in South Africa or other developing countries. Additionally, research concerning the influence of SES characteristics such as income, education and occupation, which reflect socio-economic determinants of consumers’ use of food-buying practices, is also limited. Dinkins (1997) mentions that additional behavioural research is required to determine, which factors influence consumers’ use of various money-saving methods. Harper and Crafford (2011) assert that shopping lists and use of advertisements (to plan shopping) are the two most popular pre-store food-buying practices, which is also suggested by Friedman and Rees (1988). The aforementioned pre-store food-buying practices were also identified by The Food Marketing Institute (2014) as commonly used cost-cutting methods. A majority of studies that considered the aforementioned pre-store food-buying practices were conducted in developed countries (Wilkinson and Mason, 1976 (USA); Shipchandler, 1982 (USA); Spiggle, 1987 (USA); Zaichkowsky and Sadlowsky, 1991 (USA); Thomas and Garland, 1993 (New Zealand); Polegato and Zaichkowsky, 1994 (USA); Thomas and Garland, 1996 (New Zealand); Dinkins, 1997 (USA); Putrevu and Ratchford, 1997 (USA); Block and Morwitz, 1999 (USA); Thomas and Garland, 2004 (New Zealand); Govindasamy *et al.*, 2007 (USA); Herbst and Lloyd, 2007 (USA); Bassett *et al.*, 2008 (Canada); Heinrichs *et al.*, 2011 (Germany); Mortimer and Clarke, 2011 (Australia); DeNoon, 2012 (USA); Schmidt, 2012 (Denmark); Hampson and McGoldrick’s, 2013 (UK); Mikolajczak-Degrauwe and Brengman, 2014 (Belgium); Flagg *et al.*, 2014 (USA); Zimmerman and Shimoga, 2014) (USA); Tariq *et al.*, 2016 (USA)). Furthermore, few of these inquiries considered the association of shopper and socio-demographic characteristics regarding shopping list development and/or use of advertisements among different SES groups.

Hence, an understanding of factors that account for variations in food shopping behaviour across households and SES groups in a developing country is required. The primary aims of this research are as follows: To ascertain whether there is a difference in the development of shopping lists and advertisements’ pre-store food-buying practices for planned shopping by consumers who shop in different SES areas in South Africa; to determine whether shopper characteristics have an influence on the development of shopping lists and use of advertisements as pre-store food-buying practices among consumers shopping in different SES areas in South Africa; and to establish whether socio-demographic characteristics have an impact on the development of shopping lists and use advertisements as pre-store food-buying practices among consumers shopping in different SES areas in South Africa.

## Literature

### SES

The American Psychological Association describes SES as an intersecting measurement of education, occupation, and income, which determines the social standing or class of an individual or group (American Psychological Association, 2007). Consumers’ level of income is vastly affected by their employment status. The severe recessionary conditions in the first half of 2016 were reflected in massive job losses in South Africa, and a subsequent high level of unemployment in 2016 (South African Reserve Bank, 2016). The Quarterly Labour Force Survey 2016s results show that the unemployment rate among Black and Coloured population groups is higher than other population groups, with the White population group displaying the lowest unemployment rate (Statistics South Africa, 2016b). The major differences, which are apparent among South Africans in respect of their educational levels, abilities, occupations and wealth, have resulted in one of the most unequal distributions of personal income in the world, which is clearly evident in the population groups (Petzer and De Meyer, 2013; Statistics South Africa, 2016a). Although the abolishment of Apartheid a quarter of a century ago resulted in an increase in household incomes across all population groups, there remains an unequal distribution of income. Household income directly affects a family’s ability to afford and procure food (Turrell *et al.*, 2004), and is subsequently associated with budget constraints on grocery shopping behaviours (Kim and Park, 1997).

### Pre-store food-buying practices

The primary objective of food-buying practices is to provide food shopping guidelines, which are aimed at reducing food costs, but there are also other aims, namely: to increase satisfaction with food choices; improve dietary quality; reduce shopping; obtain the best quantity and value for money spent; and make wise purchase decisions (Friedman and Rees, 1988; Herrmann and Warland, 1990). The use of food-buying practices essentially revolves around planned buying (Friedman and Rees, 1988). As consumers become more experienced, their shopping habits begin to generate a specific decision-making style (Alagöz and Ekiei, 2011). When faced with multiple decisions and numerous distractions, consumers may rely on aids (such as food-buying practices) to help simplify the decision-making process (Block and Morwitz, 1999). Pre-store planning activities are completed before the shopper enters the store, which include the following food-buying practices when planning shopping: development of a shopping list; use of advertisements; planning of menus; use of coupons; avoidance of shopping when hungry; and planning of menus around “specials” (Friedman and Rees, 1988).

It is generally only during pre-purchase and post-purchase activities that consumers experience the worth of time and money given to an activity. Consumers will decide whether or not the amount of time spent on an activity is worth its perceived monetary cost (Robinson and Nicosia, 1991). Herrington and Capella (1995) further add that, in general, shoppers tend to spend less time making a purchase, and more money in the time that is available to them. Grocery shopping is typically perceived as a time-consuming activity. Van Kenhove and De Wulf (2000) examined income and time pressure, which were applied to grocery retail shopping, to establish four grocery shopping segments: Money-poor and time-rich consumers (retired/pensioners and low education); money-poor and time-poor consumers (females, live alone or with children, employed full-time and low education levels); money-rich and time-rich consumers (older, married, retired, children left home or were about to leave, and good education); and money-rich and time-poor consumers (middle-age, live with a partner; two to three children, well-educated and full-time employed). Hence, time-pressed consumers, especially among high-income households, are generally in a hurry and are constantly looking for ways to save time. They tend to adopt time-saving strategies, which include: the purchasing of convenience food, bulk buying; shopping at less busy times; and shopping in less busy locations. They also tend to purchase fewer products than intended; make fewer unplanned purchases; and spend less time comparing product brands, prices and attributes (Davies and Madran, 1997; Chetthamrongchai and Davies, 2000; Popkowski Leszczyc *et al.*, 2004). Bawa and Ghosh (1999) shows that the higher an individual’s or household’s income, the busier they are, thus increasing the value of their time. As a result, the amount of time that the consumer is willing to spend on shopping activities often decreases. Blaylock and Smallwood (1987) suggest that consumers develop shopping lists and use advertisements as cost- and time-saving techniques. As mentioned in prior text, shopping list development and use of advertisements were identified as the two most popular pre-store food-buying practices in South Africa (Harper and Crafford, 2011) and, hence, were the primary focus of this study.

### Development of shopping lists

Bassett *et al.* (2008) defines a shopping list as “[…] itemised products to be purchased to re-stock the household […]”. Shopping lists represent the purchase intentions of consumers and are an indicator of pre-shopping planning (Spiggle, 1987). The presence of a list is associated with enhancing shopping efficiency, as it enables the shopper to remember items that are needed, avoid overbuying, organise shopping activities and control expenditure (Putrevu and Ratchford, 1997; Thomas and Garland, 2004). Gollwitzer (1993) found that specifying one’s intentions (e.g. stating or writing them down) increased the probability that the objective was achieved. This can be explained by Intons-Petersen and Fournier (1986), who suggest that the process of creating a memory aid or writing the shopping list reinforces the likelihood of remembering to purchase those specific items, regardless of whether the memory aid (shopping list) is available at the time of action, or when the person is in the grocery store. Block and Morwitz (1999) and Bassett *et al.* (2008) revealed that shoppers who used a written list perceived that it guided the shopping process. It gave them the sense of a shopping strategy, and encouraged them to shop isle by isle, thereby avoiding buying unlisted or unplanned items. Furthermore, shoppers who use a grocery list are thought to have engaged in more planning than shoppers without a list (Bassett *et al.*, 2008, p. 207). However, Thomas and Garland (1996) found that 93 per cent of consumers who used a list during a major trip purchased 2.6 times more items than those specified. They suggest that the in-store environment contributed to this additional buying. This led them to believe that the role that the written shopping list plays in grocery shopping behaviour is that of a guiding action rather than a governing action.

Thomas and Garland (2004) suggest four main list-user categories: Consumers who use a list to ensure that their shopping requirements are met; shoppers who feel that using a list simplifies the shopping experience and saves time in the shopping process; those who like to control their expenditure, stick to a budget, save and prevent overspending; and shoppers who use a list to remember “specials” and promotions, which also ensures that they obtain bargains and ultimately save money. However, Herbst and Lloyd (2007) reveal that when a shopping list is based on advertised “specials”, the use thereof leads to less spending, because consumers are less inclined to deviate from a list, which mostly comprise of items on promotion. Block and Morwitz (1999) confirm that items are more likely to be purchased upon the development of a shopping list, which increases in larger households, but is lower for products that are advertised more frequently. Schmidt (2012, p. 37) asserts that heavy advertising within a product category may lead to a higher propensity among consumers to write down brand names on shopping lists. Shipchandler (1982) disclose that consumers are more likely to use shopping lists and stock up on promoted products during recessionary periods. Thomas and Garland (1996) indicate that shopping list users spend less time shopping than non-list users, regardless of whether shopping alone or with company.

Hersey *et al.* (2001) found that low-income households are less likely to use a list when grocery shopping. Conversely, Bassett *et al.* (2008) confirm that consumers with lower household incomes are more likely to use shopping lists. Dinkins (1997) agrees that consumers who adhered to strict food budgets are less likely to use shopping lists, but more likely to have a lower education, lower household income and a larger household size, whereas population groups and gender did not have an influence. However Spiggle (1987) reports that Black consumers are more likely to write brand names on shopping lists than White consumers, but no significant difference was found for occupation, household income, age or size of household. Thomas and Garland (1993) assert that consumers with higher education are more likely to use shopping lists, whereas part- or full-time employed consumers who are less likely to use shopping lists. Gender, age, household income and marital status were found to not have an influence. DeNoon (2012) reports that men have a tendency to get lost in the supermarket without a list, so the female in the household will provide them with a list of items to purchase. Men, however, tend to only purchase specific items on the shopping list while, women are more inclined to browse. However, Polegato and Zaichkowsky (1994) determine that women used shopping list as frequently as men, whereas Blaylock and Smallwood (1987) and Bassett *et al.* (2008) confirm that women are more likely to use shopping lists. Polegato and Zaichkowsky (1994) reveal that women attach greater importance to store “specials” since they are more likely to develop the shopping list and tend to carry out household tasks that are associated with food-buying practices.

Therefore, this study aims to address the following research questions due to the dearth of research on shopping list development (dependant variable) among different SES groups (none of the aforementioned inquiries consider different SES areas) in developing countries (not one of the abovementioned studies originate from a developing country), and the divergent results in terms of shopper (independent variables) and socio-demographic (independent variables) characteristics’ association with shopping list development:

RQ1. Is there a difference in shopping list development by consumers who shop within different SES areas in South Africa?

RQ2. Do shopping characteristics have an influence on the development of shopping lists among South African consumers within different SES areas?

RQ3. Do socio-demographic factors have an impact on shopping list development among consumers within different SES areas in South Africa?

### Use of advertisements to plan shopping

Weekly newspaper advertising “specials” by grocery store chains are a major source of consumer information about food prices (Zaichkowsky and Sadlowsky, 1991; Darko *et al.*, 2013). Bassler and Newell (1982), Smith and Carsky (1996), Yoo *et al.* (2006) and East *et al.* (2008) confirm that one weekly main trip and one or more secondary ‘quick’ trips were the most common food shopping patterns among consumers. Advertising of food “specials” offers an economic incentive to make a purchase (in the form of price reductions and store coupons). Consumers may, therefore, place these advertised items on their shopping list to remember to purchase them (Block and Morwitz, 1999). According to Smith and Carsky (1996), consumers who frequently use advertisements to plan their shopping believe that this practice is highly relevant and useful in terms of saving money. Furthermore, studies have shown that there is a general lack of customer loyalty towards any specific store chains (especially during or after a recession), and that a significant number of consumers switch stores to take advantage of price discounts (Hampson and McGoldrick, 2013; Food Marketing Institute, 2014). Mikolajczak-Degrauwe and Brengman (2014) reveal that favourable attitudes towards advertising could result in impulse buying. Govindasamy *et al.* (2007) found that a majority of consumers used food advertising to plan their shopping, hence, it can be assumed that the use of this pre-store food-buying practice is quite popular and reflects a common concern for price, as is evident among South African consumers during these tough economic circumstances (National Agricultural Marketing Council, 2016).

Shipchandler (1982) and Hampson and McGoldrick’s (2013) also found consumers, especially during or after a recession, are more susceptible to advertised price discounts and made fewer shopping trips. Dinkins (1997) and Darko *et al.* (2013) established that low-income consumers are more likely and stock up on food on promotion. Zimmerman and Shimoga (2014) confirm that low-income consumers are more likely to use food advertisements than high-income consumers. Tariq *et al.* (2016) agree that consumers with higher incomes may be less price-sensitive and, hence, have less incentive to use food advertisements. However, Wilkinson and Mason (1976, p. 220) found high response rates to advertised food “specials” among low-income Black consumers and high-income White consumers. Zimmerman and Shimoga (2014) also found that food advertising had a disparate influence on different population groups. Govindasamy *et al.* (2007, p. 9) reports that consumers with higher levels of education tend to read or use food advertisements. Mortimer and Clarke (2011) reveal that female consumers consider grocery stores advertised promotional specials more important than men. Furthermore, advertised promotional specials of grocery stores were found to be more important among lower educated, older and blue collar consumers. Govindasamy *et al.* (2007) agree that older consumers are more likely to use advertisements. Zaichkowsky and Sadlowsky (1991), Thomas and Garland (2004) and Govindasamy *et al.* (2007) assert that women use advertisements more frequently than men, whereas there was no difference in terms of household income and education level. Flagg *et al.* (2014), Mittal (2016) and Tariq *et al.* (2016) agree that women use advertisements more often than men.

Hence, this research intends to address the following research questions owing to the lack of enquiry on the use of advertisements (dependent variables) among different SES groups (only one of the abovementioned studies consider different SES areas) in developing countries (none of the aforementioned inquiries originate from developing countries), and the diverse results regarding shopper (independent variables) and socio-demographic (independent variables) characteristics’ association with the use of advertisements:

RQ4. Is there a difference in the use of advertisements by consumers who shop within different SES areas in a developing country (South Africa)?

RQ5. Do consumer shopping characteristics have an impact on the use of advertisements within different SES areas in a developing country?

RQ6. Do socio-demographic factors have an influence on the use of advertisements among consumers within different SES areas in a developing country?

## Methods

### Sampling

The sample included consumers who reside in Delft, Maitland and Meadowridge in South Africa. These areas were selected for participation based on their socio-demographic and SES profiles, as provided by Statistics South Africa’s census data (Statistics South Africa, 2013a, b, c). Shoprite Usave, Shoprite, and Checkers belong to the Shoprite Holdings group and are the group’s three leading grocery store chains. Checkers offer a wide range of food and household goods for upper-income consumers and targets living standards measure (LSM) segments eight to ten (Shoprite Holdings, 2017a). Shoprite is the leading South African food retailer, which promises consumers low prices and committed customer service for a wide range of food products. Shoprite’s main customers are low-income to middle-income from the LSM segments four to seven (Shoprite Holdings, 2017b). Usave is a no-frills, small-format grocery store, which focusses on the low-income consumers from LSM segments one to five (Shoprite Holdings, 2017c). Shoprite Holdings uses similar promotional strategies for Usave, Shoprite, and Chequers, which include some television advertising, but predominantly promotional catalogues that feature “special” offers. The promotional catalogues are generally inserted in local community (free-sheets) newspapers and/or distributed directly to homes in the close proximity to the grocery stores’ geographic locations on weekly basis (Furlonger, 2015). A total of 1,200 consumers (400 in each area) who were older than 18 years anonymously and voluntarily participated in the study, which was conducted at pre-selected stores in the suburban areas, namely Usave to represent the low SES area (Delft), Shoprite to represent the middle SES area (Maitland) and Checkers to represent the high SES area (Meadowridge). A systematic sampling method was utilised with every second or third person that entered the grocery store, and who was approached to participate, according to the pace at which consumers entered the supermarket. Respondents comprised solely of volunteers who responded to an open invitation at the entrance of the supermarket and anonymity was assured.

### Design of questionnaire

Since the residents of Delft were predominantly Afrikaans speaking (Statistics South Africa, 2013a), the questionnaire was available in both English and Afrikaans to avoid any comprehension difficulties, which may have been experienced by respondents who answered questions in a second language. Possible respondents were first verified by means of pre-screening questions to establish eligibility to participate in the study. Only respondents who were primary food product buyers and decision-makers in a household and who lived in one of the SES areas (Delft, Maitland or Meadowridge) were eligible to participate in the study. The first section consisted of four questions, which provided information regarding consumers’ shopper characteristics (independent variables), namely how often consumers usually shopped for food, how long they usually took to shop for food, who usually accompanied them to the shop, as well as their means/method of payment. The second section focussed on consumers’ shopping list development and use of advertisements (dependent variables) to plan shopping as pre-store food-buying practices scales, as identified by Friedman and Rees (1988). These two scales were represented by five and six structured questions, respectively, with each question providing for four response options (1=Frequently, 2=Sometimes, 3=Seldom, and 4=Never). These options were used by Herrmann and Warland (1990) who evaluated frequencies of the use of nine food-buying practices. The third section involved the socio-demographic characteristics (independent variables) of the respondent, namely the gender, age, marital status, household size, level of education, employment status, population group and household monthly income, which were borrowed from those used in the census household questionnaire (Statistics South Africa, 2012). Written permission to conduct the research at Usave, Shoprite, and Checkers stores was sought from Shoprite Holdings. A concise consent form, incorporating the minimum essential elements, was attached to the cover page of the questionnaire, and each respondent was required to read and sign it before completing the questionnaire. Ethical approval was received from the Faculty of Applied Sciences’ Research Ethics Committee at the Cape Peninsula University of Technology.

### Data collection and analysis

The survey was conducted over five consecutive Saturdays; this day was chosen to include consumers who did not have sufficient time during the week to complete their grocery shopping. Consumers who shop on week days may have divergent shopping and socio-demographic characteristics compared to those who shop over weekends, which has been identified as a possible limitation of the study. However, Kahn and Schmittlein (1989) found that a majority of respondents indicated that they preferred to shop on a Saturday, as they had more time and could stock up for the week ahead. Two fieldworkers were employed, trained and remunerated to assist with distributing and collecting the consent forms and questionnaires, and to aid respondents, on request, to complete the questionnaire. A majority (87 per cent) of the respondents opted for the self-administered questionnaire as their chosen method to provide the requested information. A generalised linear model (GLM), using the Wald’s *χ*^2^ distribution and Bonferroni correction pairwise comparisons, was utilised to ascertain the significant differences between the consumers’ use of the two pre-store food-buying practices (dependent variables) and SES area groups, as well as the shopper and socio-demographic characteristics (independent factors).

Confirmatory factor analysis was performed to empirically test the pre-store food-buying practice scales in terms of reliability and validity. The reliability (internal consistency) of the scales were assessed using Cronbach’s *α* and composite reliability (CR) scores, which both have recommended minimum thresholds of 0.70 (Bagozzi and Yi, 2012). The Cronbach’s *α* and CR values for the shopping list development and use of advertisement scales displayed robust internal consistencies, with all of the scores exceeding 0.9 (refer to Table I). Convergent validity was evaluated by examining the average variance extracted (AVE). The AVE of the shopping list development and use of advertisement scales were both greater than 0.7 (refer to Table I), which exceeded the proposed minimum level of 0.5, and is suggestive of good convergent validity (Hair *et al.*, 2011). Discriminant validity was examined by using the square root of AVE for each scale, which should be greater than the correlation between the scales (Fornell and Larcker, 1981). The square root of AVE for the shopping list development and use of advertisements scales were 0.865 and 0.871, respectively, which was larger than the correlation score of 0.047, thereby confirming discriminant validity. Furthermore, Pearson’s correlation coefficient analysis was used to ascertain the strength of the association between variables, which revealed a predominantly positive strong (*r*>0.5) correlation for the shopping list development and use of advertisements scales, thereby indicating an overall convergence of responses (refer to Table I).

## Results and discussion

The descriptive statistics pertaining to education level, employment status and household monthly income provide a satisfactory representation of the different SES areas. A majority of the respondents in the low SES area (81.2 per cent) indicated that they had acquired a Grade 11 level of education or lower, whereas three-quarters of the middle SES area respondents specified that they had acquired a Grade 12 or lower. More than half (54 per cent) of the respondents in the high SES area indicated that they had acquired a post-matric diploma or certificate, degree or post-graduate degree. The larger part of respondents within the low (37.7 per cent), middle (55.2 per cent) and high (47.7 per cent) SES areas indicated that they were employed on a full-time basis. There was nonetheless a noticeable difference between the percentage of respondents in each area who indicated that they were unemployed (looking for work or not looking for work). The lower SES area had more (22.8 per cent) respondents compared to the middle (6.2 per cent) and high (2.0 per cent) SES areas. Furthermore, only 2 and 6.2 per cent of respondents in the low and middle SES areas specified that they were self-employed vs 14.3 per cent of respondents in the high SES area. The average monthly household in the low SES area was R800 to R3 200 for a majority (57 per cent) of respondents, whereas 77.5 per cent of the middle SES area respondents’ monthly household income was R801 to R12 800. The household monthly income for a majority (65 per cent) of respondents in the high SES area was R12 801 and above.

### Effect of SES areas on pre-store food-buying practices

The GLM showed that there were significant differences at *p*<0.001 for both the development of shopping lists (*M*=2.23, SD=1.055) and use of advertisements (*M*=1.91, SD=0.864) (dependent variables) as a result of the different SES areas (independent variable). Respondents within the low SES area (*M*=2.59, SE=0.56) displayed a lower propensity to develop shopping lists compared to the high (*M*=1.89, SE=0.51) and middle (*M*=2.22, SE=0.24) SES area respondents. The middle SES area (*M*=2.22, SE=0.24) respondents also exhibited less of an inclination to develop shopping lists in comparison to the high SES area (*M*=1.89, SE=0.51) respondents. Hence, there is a difference in shopping list development by consumers who shop within different SES areas in South Africa (*RQ1*), which exhibited a declining trend among the three SES areas. It is plausible that high SES area consumers generally have higher incomes, thereby facilitating the purchase of a larger quantity and variety of food products, which necessitates shopping lists as a memory aid (Intons-Petersen and Fournier, 1986). Dinkins (1997), Hersey *et al.* (2001) and Bassett *et al.* (2008) concur that low-income households are less likely to use a list when grocery shopping.

The opposite result was true for the use of advertisements. Respondents within the low SES area (*M*=1.57, SE=0.39) showed a higher tendency to utilise advertisements than the middle (*M*=1.83, SE=0.36) and high (*M*=2.32, SE=0.51) SES area respondents. The middle SES area (*M*=1.83, SE=0.36) respondents also displayed a higher propensity to use advertisements compared to the high SES area (*M*=2.32, SE=0.51) respondents. Consequently, there is a difference in the use of advertisements by consumers who shop within different SES areas in a developing country (*RQ4*), since the use of advertisements displayed an increasing tendency among the different SES areas. It is probable that low SES area consumers have lower incomes and, hence use advertisements to stock up on the best deals by purchasing food products that are on “special” (Dinkins, 1997; Darko *et al.*, 2013). Zimmerman and Shimoga (2014) agree that low-income consumers are more likely to use food advertisements. Tariq *et al.* (2016) confirm that consumers with higher incomes may be less price-sensitive and, therefore have less incentive to use advertisements. Furthermore, consumers in the high SES area are typically time-poor and do not have the time to consult advertisements (Van Kenhove and De Wulf, 2000).

### Influence of shopping characteristics on pre-store food-buying practices

The Bonferroni correction pairwise comparisons of estimated marginal means ascertained significant differences between shopping frequency, shopping length, co-shopping payment method (independent variables) in terms of shopping list development and/or use of advertisements (dependent variables). Therefore, consumer shopping characteristics do have an influence on pre-store food-buying practices, namely, the development of shopping lists (*RQ2*) and the use of advertisements (*RQ5*), within different SES areas in a developing country (refer to Table II).

#### Shopping frequency

Respondents in the low SES area who shopped once a week (*M*=2.62, SE=0.31) exhibited a significantly (*p*<0.05) higher predisposition to use advertisements than those who shopped for food two to three times a month (*M*=3.09, SE=0.33). A majority of the leading food retailers distribute advertisements on a weekly basis, which may have caused the higher use of advertisements by consumers who shopped once a week in the low SES area in comparison to the high SES area (Zaichkowsky and Sadlowsky, 1991; Bassler and Newell, 1982; Smith and Carsky, 1996; Yoo *et al.*, 2006; East *et al.*, 2008; Darko *et al.*, 2013). Weekly local community newspapers (free-sheets) are distributed at no cost to a majority of neighbourhoods in South Africa (Furlonger, 2015). Hence, these retail advertisements facilitate a means for consumers in the low SES area to save money by finding the best prices of food products that are on “special”. However, consumers in the high SES area displayed a much higher frequency of shopping (2-4 times a week) than the consumers in the low SES area. This result is most likely due to the lower dependence of advertised “special” offers by consumers residing in the high SES area, as they are less price sensitivity owing to their larger discretionary incomes (Tariq *et al.*, 2016). Furthermore, a majority the high SES consumers are typically time-poor and more likely to frequent the grocery stores several times a week (refer to Table II) for short shopping trips (most likely during lunchtime and/or after work to make the most of the available time) without using advertisements due to time constraints (Van Kenhove and De Wulf, 2000).

#### Shopping length

Respondents in the low SES area who indicated that they took less than half an hour to shop (*M*=3.42, SE=0.59) displayed a significantly (*p*<0.05) lower propensity to use a shopping list compared to those who indicated that they took one to two hours (*M*=2.88, SE=0.62) to shop for food. The higher development of shopping lists by consumers who spend longer periods of time shopping for groceries may be caused by longer lists of items to buy, which may be difficult to memorise, but facilitates improved budget control and a reduction of impulse purchases (Intons-Petersen and Fournier, 1986; Thomas and Garland, 2004). Furthermore, a majority of respondents in the low SES area who took less than half an hour to shop were less likely to develop a shopping list (refer to Table II), probably owing to financial constraints, which restricts them to only purchase a few essential items at a time (Frazao *et al.*, 2007). This may also cause them to buy the same types of food products or brands, which is easier to remember and requires less time in the grocery stores (Bassett *et al.*, 2008).

#### Co-shopping

Respondents in the high SES area who shopped alone (*M*=2.48, SE=0.26) or with their children/grandchildren (*M*=2.73, SE=0.29) had a significantly (*p*<0.05) lower tendency to use advertisements to plan their food shopping compared to those who shopped with their wives (*M*=1.76, SE=0.31). Co-shopping is a well-known consumer phenomenon, where shopping in groups has an influence on shopping patterns (Mangleburg *et al.*, 2004). Furthermore, the notion of pester power is another recognised consumer behaviour concept, which purports that children have an influence on parents’ and grandparents’ (adults) purchase decisions (James, 2015). Hence, consumers who shopped alone or with children/grandchildren were less likely found to be influenced by advertisements, especially in high SES areas (Tariq *et al.*, 2016). Women use advertisements more than men as confirmed in abovementioned literature (Flagg *et al.*, 2014; Mittal, 2016; Tariq *et al.*, 2016); therefore, men who shop with their wives may be influenced or guided to use advertisements to plan their shopping.

#### Payment method

Respondents in the low SES area who paid via debit card (*M*=2.52, SE=0.58) had a significantly (*p*<0.05) higher inclination to use a shopping list in comparison to those who paid by means of cash (*M*=3.19, SE=0.54). Furthermore, respondents in the low SES area who paid by means of a debit card (*M*=1.94, SE=0.30) were also found to have a significantly (*p*<0.001) higher tendency in their use of advertisements compared to those who paid via credit card (*M*=3.16, SE=0.49) or cheque (*M*=3.94, SE=0.51). However, few respondents in the low SES area used credit cards and cheques as payment methods. The growing use of debit cards as a payment method frequently results in larger purchases, as consumers are not limited to the amount of cash that they have in their possession. Borzekowski and Kiser (2008) and Klee (2008) assert that the probability of consumers using a debit card increases as their income increases. Furthermore, carrying large sums of cash in low SES areas poses an increased security risk (Arango and Taylor, 2009). Hence, debit cards enable consumers in the low SES area to purchase food in bulk and a larger variety of items, which may increase their use of advertisements and shopping list development.

### Influence of socio-demographic characteristics on pre-store food-buying practices

No significant differences were determined for age, marital status and education level for both pre-store food-buying practices in any of the SES areas. However, the Bonferroni correction pairwise comparisons of estimated marginal means determined significant differences between gender, household numbers, employment status, population group and household monthly income (independent variables) regarding the development of shopping lists and/or use of advertisements (dependent variables). Therefore, consumer socio-demographic characteristics do have an impact on pre-store food-buying practices, namely the development of shopping (*RQ3*) and the use of advertisements (*RQ6*), within different SES areas in South Africa (refer to Table III).

#### Gender

Female respondents (*M*=1.88, SE=0.28) in the high SES area displayed a significantly (*p*<0.05) larger propensity to use a shopping list compared to male respondents (*M*=2.17, SE=0.30). Furthermore, female respondents (*M*=1.43, SE=0.25) in the middle SES area also showed a significantly (*p*<0.001) larger inclination to use advertisements as a pre-store food-buying practice than male respondents (*M*=1.85, SE=0.32). Females typically have the primary responsibility of shopping for food products in a developing country, hence they frequently utilise a variety of pre-store food-buying practices (such as the use of advertisements and shopping list development) more frequently than males (Flagg *et al.*, 2014; Mittal, 2016; Tariq *et al.*, 2016), irrespective of the SES area, as confirmed by the results of this study.

#### Household numbers

Households with two members (*M*=2.35, SE=0.28) in the high SES area exhibited a significantly (*p*<0.05) lower propensity to use advertisements in comparison to households with five members (*M*=1.80, SE=0.30). Larger households generally have a larger need to employ a variety of pre-store food-buying practices to save money than smaller households, which were found to use advertisements less frequently. Dinkins (1997) ascertained that consumers who adhered to strict food budgets were more likely to have larger household sizes. This can be attributed to the fact that household grocery expenditure increases with family size (Tariq *et al.*, 2016). Murthi and Rao (2012) agree that large families tended to evaluate prices, for example, via advertisements, as a means to save money more often than small families.

#### Employment status

Self-employed respondents (*M*=2.28, SE=0.30) in the high SES area showed a significantly (*p*<0.001) lower tendency to use a shopping list in comparison to pensioner/retired (*M*=1.46, SE=0.32) respondents. Generally, senior citizens in South Africa have lower incomes, more time to plan, and their memories may not be as good compared to their younger self-employed counterparts. Putrevu and Ratchford (1997) and Thomas and Garland (2004) confirm that shopping lists enable shoppers to remember items and avoid overbuying.

#### Population group

White respondents (*M*=4.00, SE=0.64) in the low SES area showed a significantly (*p*<0.05) lower predisposition to use advertisements compared to Black (*M*=2.17, SE=0.23) and Coloured (*M*=2.20, SE=0.22) respondents. The higher lower use of advertisements by White consumers may be as a result of higher disposable incomes, thereby enabling the purchase of a larger variety and quantity of food products owing to low levels of price sensitivity and greater time constraints (Govindasamy *et al.*, 2007; Tariq *et al.*, 2016). Hence, households in low SES areas (mainly Black and Coloured consumers in South Africa) use advertisements more, mainly in a bid to save money by buying food products that are on “special” (Hersey *et al.*, 2001; Govindasamy *et al.*, 2007; Tariq *et al.*, 2016).

#### Household monthly income

Respondents in the high SES area with a household monthly income of R25 601 ($1 970)-R51 200 ($3 938) (*M*=2.41, SE=0.29) displayed a significantly (*p*<0.05) lower inclination to make use of advertisements compared to those with a monthly income of R801 ($67)-R 3 200 ($246) (*M*=1.77, SE = 0.31) and R3 201 ($247)-R6 400 ($492) (*M*=1.87, SE=0.30). Respondents in the middle SES area with a household monthly income of R25 601 ($1 970)-R51 200 ($3 938) (*M*=1.94, SE=0.29) also exhibited a significantly (*p*<0.05) lower tendency to make use of advertisements compared to those with a monthly income of R801 ($67)-R 3 200 ($246) (*M*=1.38, SE=0.26) and R3 201 ($247)-R6 400 ($492) (*M*=1.45, SE=0.26). The low usage of advertisements by respondents with high incomes in the high and middle SES areas could be explained by the fact that these consumers do not need to save money, but probably have time constraints. These consumers are characteristically money-rich and time-poor (Van Kenhove and De Wulf, 2000) and, therefore do not have the time or the need to use advertisements. Mittal (2016) and Tariq *et al.* (2016) concur that consumers with high levels of education and income may be less price-sensitive and, therefore less likely to use advertisements than their lower income counterparts.

## Implications and conclusions

Shopping list development and the use of advertisements have been broadly researched over several decades in developed countries. However, little academic research has been conducted in terms of the pre-store food-buying practices in developing countries. Furthermore, few studies consider pre-store food-buying practices in different SES areas from a developed and developing country perspective. Additionally, there is a dearth of research on the association of shopping and socio-economic characteristics with the development of shopping lists and use of advertisements in different SES areas in emerging and developed nations. Several of the independent variables have not previously been investigated in relation to the abovementioned pre-store food-buying practices. Therefore, this study makes a valuable contribution to theory development and food-buying practice research in a developing country. The results of this research could also help consumers in developing countries with food-buying practices to reduce shopping and food costs, as well as make more prudent shopping decisions. This study also provides international supermarket chains with insight into the food-buying practices, shopping and socio-demographic characteristics of South African consumers, which provides them with valuable information to diversify their operations into developing country markets.

Grocery stores should continue to disseminate weekly advertisements, which conspicuously display their food products to attract consumers into their stores, but also encourage children to accompany their parents/grandparents by creating a child friendly shopping atmosphere and environments, which may have a favourable effect on turnover. Furthermore, consumers could be enticed to stay in the supermarkets for longer periods of time via promotional offers, which will have a positive effect on sales. Grocery stores should create a senior citizen friendly shopping atmosphere and environment (e.g. a day where pensioners receive a special promotional discount) that will entice these lucrative shoppers to frequent their outlets. Furthermore, the higher usage of advertisements by Black and Coloured consumers provides discerning grocery stores with the prospect of conspicuously featuring their food products in the promotional catalogues that are directed at these consumer groups. Larger households should be targeted with bulk food and other sales promotions that are featured in promotional catalogues, which should also be distributed to consumers in low-income areas due to their high incidence of advertisement usage.

It can be concluded that high SES area consumers have the highest propensity of shopping list development, and low SES area consumers show the highest incidence of advertisement usage in a developing country, which is analogous to the findings in developed countries. Grocery stores could place emphasis on their marketing communications by highlighting the need to add certain food products to shopping lists. For example, grocery stores could create a mobile app to facilitate shopping list development when targeting these consumers due to the rapid growth and adoption of mobile devices by South Africans residing in all SES areas. Mobile connections exceed the South African population since many consumers use multiple mobile devices (Goosen, 2017). Grocery stores should persist with promotional catalogues, which are distributed to consumers in low SES areas. Furthermore, the mobile app could be used to disseminate promotional offers directly to these consumers, which enables consumers to add advertised food products directly to their shopping list, thereby increasing their use of advertisements and shopping list development in a developing country.

## Figures and Tables

**Table I:** Pre-store food-buying practices (shopping lists development and use of advertisements) descriptive statistics, confirmatory factor analysis (factor loadings), Cronbach’s α, CR, AVE and Pearson’s correlation

							Pearson’s correlation					
Pre-store food-buying practices	*M*	SD	Factor loadings	AVE	CR	Cron. *α*	1	2	3	4	5	6
*Shopping list development*												
Write down a list (1)	2.40	1.347	0.952	0.748	0.936	0.915	1.000					
Having a list while shopping (2)	2.39	1.328	0.946				0.948*	1.000				
Purchase according to a list (3)	2.43	1.312	0.935				0.941*	0.923*	1.000			
Check at home first (4)	2.04	1.009	0.794				0.647*	0.639*	0.613*	1.000		
Know exactly what to buy (5)	1.90	1.072	0.657				0.483*	0.480*	0.456*	0.598*	1.000	
*Use of advertisements*												
Look for advertisements (1)	1.63	1.005	0.947	0.758	0.949	0.935	1.000					
Plan to shop for advertised “specials” (2)	1.72	1.011	0.925				0.906*	1.000				
Pay attention to advertisements (3)	1.70	0.990	0.925				0.898*	0.896*	1.000			
Shop knowing about “specials” (4)	1.95	0.948	0.846				0.768*	0.722*	0.748*	1.000		
Use advertisements to plan shopping (5)	2.15	0.972	0.802				0.680*	0.653*	0.637*	0.617*	1.000	
Immediately plan to shop once aware of a “special” (6)	2.30	1.041	0.762				0.646*	0.606*	0.602*	0.554*	0.654*	1.000

**Note:** *Correlation is significant at the 0.01 level (two-tailed)

**Table II:** Influence of shopping characteristics on pre-store food-buying practices – development of shopping lists (SL) and use of advertisements (Ad)

	High SES area (*n*=400)			Middle SES area (*n*=400)			Low SES area (*n*=400)		
Shopper characteristics	*n*	%	*p*	*n*	%	*p*	*n*	%	*p*
*Shopping frequency*									
Every day (1)	87	21.7	0.990 (SL) 0.327 (Ad)	94	23.5	0.631 (SL) 0.233 (Ad)	91	22.7	0.632 (SL) 0.032** (Ad) (3)-(4)^B^
2-4 times a week (2)	137	34.2		103	25.8		78	19.5	
Once a week (3)	132	33.0		141	35.2		194	48.5	
2-3 times a month (4)	21	5.3		31	7.7		16	4.0	
Once a month (5)	23	5.8		31	7.8		21	5.3	
*Shopping length*									
Less than half an hour (1)	169	42.2	0.949 (SL) 0.915 (Ad)	162	40.5	0.205 (SL) 0.061 (Ad)	207	51.8	0.041** (SL) (2)-(1)^B^ 0.561 (Ad)
.5-1 hour (2)	165	41.3		181	45.2		151	37.7	
1-2 hours (3)	56	14.0		49	12.3		30	7.5	
More than 2 hours (4)	10	2.5		8	2.0		12	3.0	
*Co-shopping*									
Shop alone (1)	282	70.5	0.931 (SL) 0.004** (Ad) (3) – (1 & 5)^b^	265	66.2	0.464 (SL) 0.672 (Ad)	280	70.0	0.388 (SL) 0.106 (Ad)
Husband (2)	29	7.2		34	8.5		24	6.0	
Wife (3)	31	7.7		38	9.5		6	1.5	
Partner (4)	3	0.8		3	0.7		6	1.5	
Children/grandchildren (5)	25	6.2		31	7.8		64	16.0	
Relative(s) (6)	10	2.5		11	2.8		10	2.5	
Friend(s) (7)	6	1.5		6	1.5		9	2.2	
Colleague(s ) (8)	1	0.3		0	0.0		0	0.0	
Family (9)	13	3.3		12	3.0		1	0.3	
*Payment method*									
Cash (1)	147	36.8	0.513 (SL) 0.586 (Ad)	282	70.5	0.144 (SL) 0.677 (Ad)	372	93.0	0.034** (SL) (2)-(1)^b^ 0.000* (Ad) (2)-(3 & 4)^a^
Debit card (2)	173	43.2		112	28.0		24	6.0	
Credit card (3)	68	17.0		3	0.7		2	0.5	
Cheque (4)	6	1.5		1	0.3		2	0.5	
Cape consumers (buy aid) (5)	6	1.5		2	0.5		0	0.0	

**Notes:** *Wald *χ*^2^ test showed a significant difference at *p*<0.001; **Wald *χ*^2^ test showed a significant difference at *p*<0.05. ^a^Bonferroni correction pairwise comparisons mean difference is significant at the 0.001 level; ^b^Bonferroni correction pairwise comparisons mean difference is significant at the 0.05 level

**Table III:** Influence socio-demographic characteristics on pre-store food-buying practices – development of shopping lists (SL) and use of advertisements (Ad)

	High SES area (*n*=400)			Middle SES area (*n*=400)			Low SES area (*n*=400)		
Socio-demographic characteristics	*n*	%	*p*	*n*	%	*p*	*n*	%	*p*
*Gender*									
Male (1)	124	31.0	0.017** (SL) (2)-(1)^B^ 0.115 (Ad)	130	32.5	0.455 (SL) 0.001* (Ad) (2)-(1)^A^	61	15.2	0.900 (SL) 0.312 (Ad)
Female (2)	276	69.0		270	67.5		339	84.8	
*Age (years)*									
18-25 (1)	22	5.5	0.144 (SL) 0.368 (Ad)	21	5.3	0.072 (SL) 0.872 (Ad)	38	9.5	0.988 (SL) 0.662 (Ad)
26-35 (2)	52	13.0		99	24.7		95	23.7	
36-45 (3)	72	18.0		95	23.7		58	14.5	
46-55 (4)	90	22.5		87	21.8		141	35.2	
56-65 (5)	83	20.7		49	12.2		59	14.8	
>66 (6)	81	20.3		49	12.3		9	2.3	
*Marital status*									
Married (1)	225	56.2	0.088 (SL) 0.158 (Ad)	210	52.5	0.304 (SL) 0.561 (Ad)	219	54.8	0.964 (SL) 0.361 (Ad)
Living together (2)	23	5.7		26	6.5		14	3.5	
Single (3)	73	18.3		96	24.0		89	22.2	
Widower/widow (4)	31	7.7		38	9.5		38	9.5	
Separated (5)	3	0.8		0	0.0		2	0.5	
Divorced (6)	45	11.3		30	7.5		38	9.5	
*Household (HH) numbers*									
1 (1)	57	14.2	0.511 (SL) 0.018** (Ad) (5)-(2)^B^	41	10.2	0.876 (SL) 0.553 (Ad)	2	0.5	0.864 (SL) 0.537 (Ad)
2 (2)	121	30.3		66	16.5		31	7.7	
3 (3)	92	23.0		88	22.0		84	21.0	
4 (4)	73	18.2		100	25.0		100	25.0	
5 (5)	41	10.3		42	10.5		74	18.5	
6+(6)	16	4.0		63	15.8		109	27.3	
*Education level*									
Grade 1-7 (1)	7	1.7	0.579 (SL) 0.861 (Ad)	40	10.0	0.085 (SL) 0.256 (Ad)	84	21.0	0.858 (SL) 0.984 (Ad)
Grade 8-11 (2)	56	14.0		155	38.7		241	60.2	
Grade 12 (3)	121	30.3		107	26.7		68	17.0	
Post-matric diploma or certificate (4)	102	25.5		58	14.5		2	0.5	
Degree (5)	65	16.2		33	8.3		0	0.0	
Post-graduate degree (6)	49	12.3		7	1.8		5	1.3	
*Employment status*									
Employed (full-time) (1)	191	47.7	0.001* (SL) (7)-(3)^a^ 0.087 (Ad)	221	55.2	0.565 (SL) 0.161 (Ad)	151	37.7	0.925 (SL) 0.770 (Ad)
Employed (part-time) (2)	27	6.7		26	6.5		29	7.3	
Self-employed (3)	57	14.3		20	5.1		8	2.0	
Unemployed (looking for work) (4)	5	1.3		18	4.5		50	12.5	
Unemployed (not looking for work) (5)	3	0.7		7	1.7		41	10.3	
Housewife/homemaker (6)	24	6.0		31	7.8		74	18.5	
Pensioner/retired (7)	86	21.5		67	16.7		34	8.5	
Student (8)	7	1.8		6	1.5		8	2.0	
Not working – other (9)	0	0.0		4	1.0		5	1.2	
*Population group*									
Black African (1)	24	6.0	0.150 (SL) 0.112 (Ad)	70	17.5	0.956 (SL) 0.051 (Ad)	40	10.0	0.517 (SL) 0.002** (Ad) (1 & 2)-(4)^b^
Coloured (2)	134	33.5		312	78.0		358	89.5	
Indian/Asian (3)	11	2.8		3	0.8		1	0.2	
White (4)	227	56.7		10	2.5		1	0.3	
Other (5)	4	1.0		5	1.2		0	0.0	
*Household monthly income (R1=$1)*									
Less than R800 ($67) (1)	2	0.5	0.853 (SL) 0.044** (Ad) (2 & 3)-(6)^B^	9	2.3	0.510 (SL) 0.033** (Ad) (2 & 3)-(6)^B^	52	13.0	0.376 (SL) 0.266 (Ad)
R801 ($67)-R3 200 ($246) (2)	28	7.0		100	25.0		228	57.0	
R3 201 ($247)-R6 400 ($492) (3)	43	10.7		116	29.0		82	20.5	
R6 401 ($493)-R12 800 ($985) (4)	67	16.8		94	23.5		29	7.2	
R12 801 ($986)-R25 600 ($1 969) (5)	115	28.7		61	15.2		7	1.8	
R25 601 ($1 970)-R51 200 ($3 938) (6)	86	21.5		17	4.2		2	0.5	
R51 201+ ($3 938+) (7)	59	14.8		3	0.8		0	0.0	

**Notes:** *Wald *χ*^2^ test showed a significant difference at *p*<0.001; **Wald *χ*^2^ test showed a significant difference at *p*<0.05. ^a^Bonferroni correction pairwise comparisons mean difference is significant at the 0.001 level; ^b^Bonferroni correction pairwise comparisons mean difference is significant at the 0.05 level
